# Interrupted aortic arch associated with aortic coarctation, bicuspid aortic valve and moderate to severe mitral valve regurgitation in an adult patient: a case report

**DOI:** 10.11604/pamj.2022.43.149.36281

**Published:** 2022-11-22

**Authors:** Othmane Haddani, Besart Cuko, Frederic Vanden Eynden

**Affiliations:** 1 Department of Cardiac Surgery, Erasme University Hospital, Université Libre de Bruxelles, Brussels, Belgium,; 2 Department of Cardiology and Cardio-Vascular Surgery, Cardiologic Hospital of Haut-Lévèque, Bordeaux University Hospital, Pessac, France

**Keywords:** Aortic arch interruption, aortic coarctation, bicuspid aortic valve, congenital abnormality, case report

## Abstract

Interrupted aortic arch is a rare congenital abnormality with a high mortality rate in infancy conditioning only a few cases reported in adult patients. The principal finding is a complete loss of continuity between the ascending and descending portions of aorta, and is usually associated with other cardiac defects. In this case report, we present a 22-year-old male patient with refractory hypertension and diagnosis of interrupted aortic arch associated with aortic coarctation, bicuspid aortic valve and moderate to severe mitral valve regurgitation. We decided to perform a surgical correction and the patient underwent to bypass grafting of the ascending-to-descending aorta, and mitral valve repair. Interrupted aortic arch must be considered in the differential diagnosis of adult patient with refractory hypertension and a careful physical examination is crucial for ensuring the correct diagnosis of rare congenital abnormality non made until adulthood.

## Introduction

Interrupted aortic arch (IAA) is a rare congenital abnormality with a high mortality rate in infancy defined as a complete loss of continuity between the ascending and descending portions of aorta. In most of the cases IAA is associated with additional anatomical cardiac defects [1]. Survival without surgical reparation is rarely and is relied on the development and adaptation of an extensive arterial collateral network, which is essential for the maintenance of distal blood flow. In the medical literature only a few cases of IAA in adult are reported. The diagnosis should be suspected in the presence of refractory hypertension and a differential between upper and lower limb blood pressure [2]. The authors describe an unusual case of a 22-year-old male patient in whom the diagnosis of IAA was not made until adulthood and which was successfully corrected surgically.

## Patient and observation

**Patient information:** a 22-year-old military male with a medical history of refractory arterial hypertension in medical treatment with Angiotensin-converting enzyme inhibitor was hospitalized in Algeria with sudden dyspnea, bilateral lower extremity claudication and palpitations.

**Clinical findings:** on physical examination the patient had shortness of breath and sudoresis. Asymmetric blood pressure between right arm and left arm, respectively 130/80 mmHg and 100/80 mmHg, and a differential between upper and lower limb blood pressure was revealed with a lower limb blood pressure of 90/70 mmHg on both lower extremities. Cardiac auscultation revealed an important systolic murmur throughout the entire cardiac region irradiating to the right carotid artery. Pulmonary auscultation was normal and peripheral oxygen saturation (in room air) was 99-100%.

**Figure 1:**
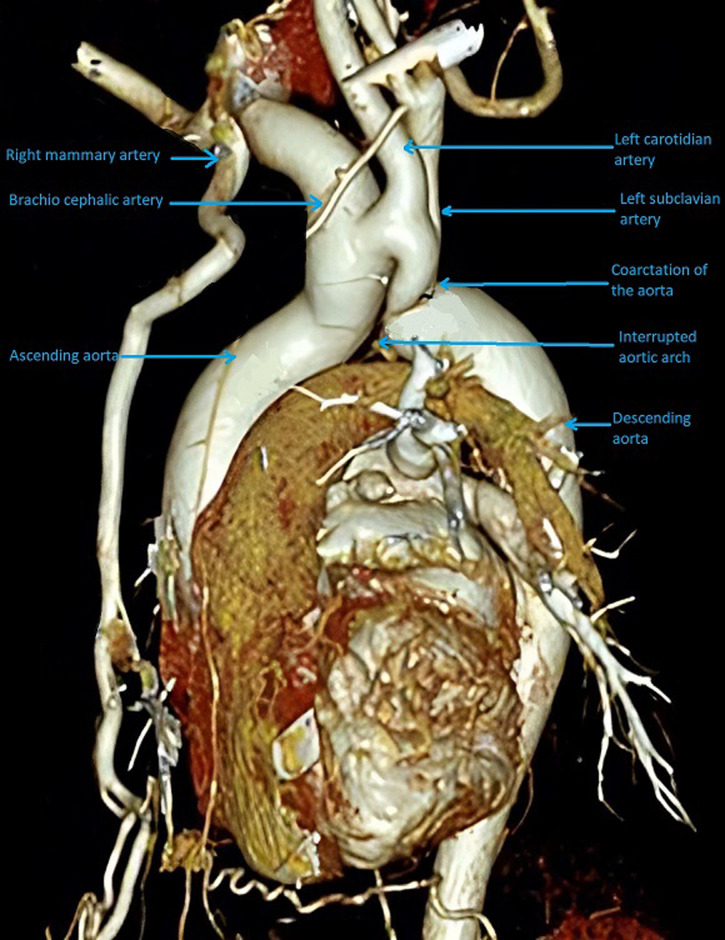
pre-operative computed tomography angiography (CTA) reconstruction

**Diagnostic assessment:** the 12-lead electrocardiogram showed normal sinus rhythm. Admission laboratory studies showed no significant changes, including myocardial necrosis biomarkers. Transthoracic echocardiography revealed aortic coarctation, bicuspid aortic valve without stenosis or insufficiency associated and a moderate to severe mitral valve insufficiency due to anterior leaflet prolapse. Computed tomography angiography (CTA) demonstrated a type C aortic arch interruption immediately after the brachiocephalic trunk, aortic coarctation with small-sized left subclavian and left carotid arteries and a huge-sized brachiocephalic trunk with a communication towards the left carotid artery (Figure 1).

**Diagnosis:** type C interrupted aortic arch associated with aortic coarctation, bicuspid aortic valve and moderate to severe mitral valve regurgitation.

### Therapeutic interventions

**Figure 2:**
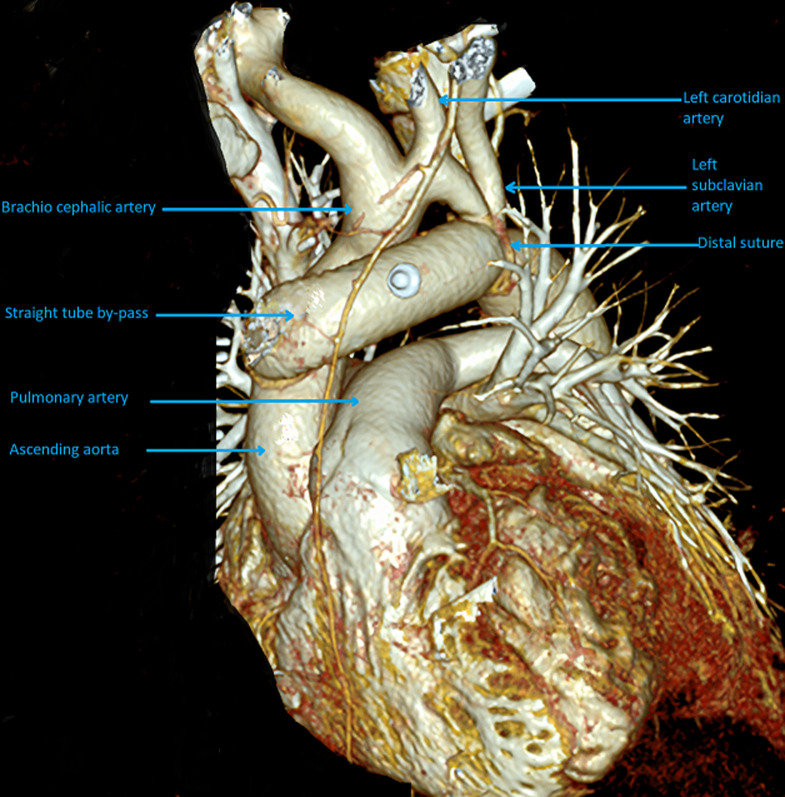
post-operative computed tomography angiography (CTA) reconstruction

The patient was transferred to our Cardiac Surgery Unit in Belgium for surgical intervention. Under general anesthesia a full sternotomy was performed. The cardiopulmonary bypass (CPB) was established by the right axillary artery and bicaval venous cannulation. During the patient´s cooling isolation of the supra-aortic trunks was performed. At the target temperature of 26°C general circulatory arrest was started and correction of the aortic arch with the interposition of 30 mm straight vascular Dacron tube between the ascending aorta and the descending portions of aorta after coarctation was performed (Figure 2). After restoration of general blood circulatory mitral valve repair by insertion of annuloplasty ring was performed. Postprocedural recovery was uneventful with a good hemodynamic response. At the end of operation, right arm and left arm blood pressure was equal as also the upper and lower limbs blood pressures were equal with non-representative differences.

**Follow-up and outcome of intervention:** the patient recovered uneventfully with no complications and was discharged at home after 10 days. Physical examination before discharge finds no difference on blood pressure between all extremities. Transthoracic echocardiography showed excellent surgical resultants of aortic arch correction and mitral valve repair.

**Informed consent:** patient´s informed consent for the procedure and for data collection was obtained.

## Discussion

IAA is a rare congenital cardiovascular malformation and severe congestive heart failure is the major presentation of IAA in infants [3]. It is defined as a complete loss of continuity between the ascending and descending portions of aorta and in most of the cases IAA is associated with additional anatomical cardiac defects [1]. First IAA was described by Steidele in 1778 and Celoria *et al*. introduced the first classification system in 1959, which is almost still universally used [4]. In this classification, IAA is divided in three different types according to the site of aortic interruption: type A if the discontinuity is distal to the origin of the left subclavian artery (LSA), type B if the discontinuity is located between the left common carotid artery (LCCA) and the LSA, and type C between the innominate artery and the LCCA. Sakellaridis *et al*. reported the following rates: type A 79%; type B 16% and type C 3% [5].

Almost all cases of IAA are diagnosticated in the neonatal period with a mortality rate of 90% in the first year, the majority in the first few days, if untreated [6]. Survival into adulthood without treatment relies on the development of an extensive collateral network, essential for the maintenance of distal flow. Clinical presentation in adult varies from absence of symptoms to refractory hypertension, differential blood pressure, claudication, headache, malaise, and congestive heart failure. Physical examination is crucial to detect pathological murmurs and the upper to lower limbs blood pressure differential. Transthoracic echocardiography is the technique of choice for diagnosis, despite some limitations in evaluating the aortic arch and the descending aorta. In addition, CTA and magnetic resonance imaging are useful and may be particularly beneficial in the evaluation of a suspected IAA [7,8].

According to the literature, surgical correction is the preferred treatment for the IAA as surgery is successful in most patients [9]. Surgical correction can be performed as extra-anatomic bypass with a interposition of a vascular tube graft or as intra-anatomic bypass with an end-to-end anastomosis, depending upon the anatomy and the location of the aortic discontinuity. In adult the extra-anatomic correction technique, followed by percutaneous stent coarctation correction, is the most frequently described surgical approach [10].

Our patient underwent intra-anatomic end-to-end approach with the interposition of 30 mm straight vascular Dacron tube between the ascending aorta and the descending portions of aorta after coarctation. We chosed this strategy for total one-stage anatomical correction as we also performed a mitral valve repair by insertion of annuloplasty ring. Postprocedural recovery was uneventful, and the patient was discharged at home totally asymptomatic.

## Conclusion

Type C interrupted aortic arch associated with aortic coarctation, bicuspid aortic valve and moderate to severe mitral valve regurgitation in an adult patient is a rare condition. Care should be taken, and a physical examination is crucial in presence of refractory hypertension and the upper to lower limbs differential blood pressure. Surgical correction is the preferred treatment for the IAA as surgery is successful in most patients.
